# Editorial: LncRNA and their role on epigenome in cancer

**DOI:** 10.3389/fmolb.2024.1533057

**Published:** 2024-12-04

**Authors:** Qi Su, Francesca Maria Orlandella, Giovanni Smaldone, Yitao Qi

**Affiliations:** ^1^ Key Laboratory of the Ministry of Education for Medicinal Resources and Natural Pharmaceutical Chemistry, National Engineering Laboratory for Resource Developing of Endangered Chinese Crude Drugs in Northwest China, College of Life Sciences, Shaanxi Normal University, Xi’an, Shaanxi, China; ^2^ University of Naples Parthenope, Naples, Italy; ^3^ IRCCS SYNLAB SDN, Naples, Italy

**Keywords:** lncRNA, cancer, gene therapy, gene editing technology, biomarkers, epigenetic

In this Research Topic “LncRNA and their role on epigenome in cancer”, a series of original comprehensive studies and reviews were collected, that describe the critical role of LncRNA (long non-coding RNA) in the regulation of cancer. LncRNAs refer to non-protein-coding RNA molecules with a length of more than 200 bp (Srinivas et al., 2024; Zhang, 2024). More studies have shown that LncRNAs play an important role in the regulation of gene expression at the epigenetic, transcriptional, and post-transcriptional levels (Luo et al., 2021; Yang et al., 2022), and are closely associated with the occurrence and development of cancer (Toden et al., 2021). Therefore, LncRNAs are likely to become a potential target for clinical treatment of cancer, and the establishment of LncRNA based therapy has broad prospects in exploring the pathogenesis of cancer. Besides their therapeutic potential, the knowledgement of circulating LncRNAs could be crucial for the develop of novel minimally invasive diagnostic and prognostic strategy in cancer patients since, when are released in the blood, they are stable and free from nuclease degradation. In view of the important regulatory role of LncRNA in cancer and the latest research progress in this field, we organized this study to comprehensively summarize the new mechanisms, potential and obstacles of LncRNA in regulating tumor development. The following is the summary of the original research and reviews from each contributor for this Research Topic of “LncRNA and their role on epigenome in cancer.”

In the first article in Research Topic, Chi et al. used medical big data and artificial intelligence to construct a prediction model of disulfidptosis response-related LncRNA on the TCGA-COAD cohort, and identified that disulfidptosis response-related LncRNA were a robust predictors of colon adenocarcinoma (COAD). The model facilitated the precise classification of clinical COAD patients and identified specific subgroups that are more beneficial to immunotherapy and chemotherapy, providing favorable information for the development of personalized treatment strategies for COAD patients. In this study, these LncRNAs were evaluated as independent prognostic factors for COAD, and patients in the low-risk group showed higher overall survival, providing new insights into immunotherapy response and prognosis assessment in COAD. In conclusion, this study successfully identified specific LncRNAs that are closely associated with death by disulfide, revealing new prognostic biomarkers and potential therapeutic targets of COAD.

In the next article, Moaveni et al. provide a comprehensive and critical analysis of recent advances and challenges in gene therapy for pediatric cancer. The challenges facing pediatric cancer are more unique and complex than those facing adult cancers. This review elucidates current innovative systems that promise to address pediatric tumors, such as viral platforms (retroviruses, lentiviruses, adeno-associated viruses), non-viral platforms (liposomes, nanoparticles), and immunotherapies (CAR T-cell therapy, tumor-targeting antibodies, checkpoint inhibitors), and discussed the barriers and challenges posed by pediatric cancer to gene therapy. In addition, the authors conduct a comprehensive analysis of ethical and clinical model studies to provide valuable insights into gene therapy for pediatric cancer. In summary, this review summarizes the latest overview of gene therapy in pediatric cancer, highlights rapid scientific progress and substantial barriers to be addressed, and proposes gene therapy as a treatment available to pediatric patients worldwide.

In the third article in this Research Topic, Wei et al. reviewed the differential expression and clinical significance of LncRNA in the development and progression of lung adenocarcinoma (LUAD). LncRNA can regulate the occurrence and development of tumors, and are considered as one of the research hotspots in human LUAD and other malignant tumors. The authors first introduced the function and research status of LncRNA, and summarized the regulatory mechanism of LncRNA in LUAD and the influence of differential expression of LncRNA on the progress of LUAD. Subsequently, the authors described the signal transduction pathways of LncRNA involved in LUAD, including JAK/STAT, PI3K/Akt/mTOR, Wnt/β-catenin, TGF-β/SMAD, and ATR/CHK1 signaling pathways and miRNA signaling axis. A comprehensive summary of the close relationship between LncRNA and LUAD provides a novel idea for the early diagnosis and treatment of LUAD.

Glutathione peroxidase 4 (GPX4) is a unique antioxidant enzyme that can protect cells from membrane lipid peroxidation and maintain redox homeostasis, and its role in patients with hepatitis B virus-associated acute-on-chronic liver failure (HBV-ACLF) was studied (Su et al.). In 289 participants, GPX4 expression levels in serum and peripheral blood mononuclear cells (PBMCs) of HBV-ACLF patients were lower than in non-HBV ACLF patients, chronic hepatitis B (CHB) and healthy control (HC) individuals. At the same time, in HBV-ACLF patients, the methylation level of GPX4 promoter is higher, and it is related to oxidative stress and inflammation related molecules. The methylation level of the GPX4 promoter, as assessed by the ROC curve, was identified as a biomarker for predicting 90-day mortality in HBV-ACLF patients. This study shows the importance of GPX4 in HBV-ACLF, which provides new ideas for the clinical treatment and prognosis of HBV-ACLF, and the specific regulatory mechanism needs to be further explored.

Finally, in this Research Topic, based on the correlation between the basement membrane and related LncRNA and the prognosis of head and neck squamous cell carcinoma (HNSCC), Bu et al. first used the Cancer Genome Atlas (TCGA) database to obtain HNSCC related data. In this study, differentially expressed LncRNAs were screened, and a basement membrane LncRNA-based prognostic model was successfully established and comprehensively analyzed from different perspectives. By establishing a risk model, 14 pairs of basement membrane LncRNA were evaluated comprehensively, indicating that risk assessment can be used as a reliable prognostic factor. If *in vitro* and *in vivo* studies can be combined at a later stage, it will be more helpful to explore new targets for head and neck squamous cell carcinoma (HNSCC) treatment, and provide clinical guidance for clinical research and drug development.

The incidence and mortality of cancer are increasing year by year, and it has become the main cause threatening human life and health worldwide (He et al., 2023; Schwartz, 2024). In recent years, gene therapy approaches have become a new way of clinical treatment of cancer, such as CRISPR system has been widely used in cancer-related basic research (Schambach et al., 2024). Targeting gene mutations that drive tumor growth and spread provide new possibilities for the development of more effective and personalized cancer treatment (Chehelgerdi et al., 2024). With the application of gene editing technology, a large number of LncRNA have been found to play important regulatory roles in cancer, including participating in chromatin modification, genomic imprinting, and intranuclear transport (Kopp and Mendell, 2018; Liu et al., 2024). In addition, immunotherapy and other methods by regulating DNA levels are gradually being developed. In conclusion, this Research Topic confirmed that the differential expression of LncRNA is closely related to the development of cancer, but the specific regulatory mechanism of LncRNA in cancer has been studied with further challenges. Therefore, as the potential mechanisms of LncRNA in the occurrence and development of cancer continue to be explored, future studies are expected to develop various cancer-related LncRNA, providing new targets for the early diagnosis and clinical treatment of cancer.
